# Puumala Hantavirus Excretion Kinetics in Bank Voles (*Myodes glareolus*) 

**DOI:** 10.3201/eid1408.080221

**Published:** 2008-08

**Authors:** Jonas Hardestam, Malin Karlsson, Kerstin I. Falk, Gert Olsson, Jonas Klingström, Åke Lundkvist

**Affiliations:** * Swedish Institute for Infectious Disease Control, Solna, Sweden; † Karolinska Institutet, Stockholm, Sweden; ‡ Swedish Defence Research Agency, Umeå, Sweden; § Swedish University of Agricultural Sciences, Umeå; 1 These authors contributed equally to this article.

**Keywords:** Puumala hantavirus, bank vole, zoonosis, saliva, urine, feces, real-time RT-PCR, nephropathia epidemica, transmission, shedding, research

## Abstract

Puumala hantavirus is present in bank voles (*Myodes glareolus*) and is believed to be spread mainly by contaminated excretions. In this study, we subcutaneously inoculated 10 bank voles with Puumala virus and sampled excretions until day 133 postinfection. Levels of shed viral RNA peaked within 11–28, 14–21, and 11–28 days postinfection for saliva, urine, and feces, respectively. The latest detection of viral RNA was 84, 44, and 44 days postinfection in saliva, urine, and feces, respectively. In contrast, blood of 5 of 6 animals contained viral RNA at day 133 postinfection, suggesting that bank voles secrete virus only during a limited time of the infection. Intranasal inoculations with bank vole saliva, urine, or feces were all infectious for virus-negative bank voles, indicating that these 3 transmission routes may occur in nature and that rodent saliva might play a role in transmission to humans.

## Abstract 2

One-sentence summary for table of contents: Virus may be transmitted by saliva, urine, and feces, and saliva may play a role in transmission to humans.

*Hantavirus*, a genus within the family *Bunyaviridae*, contains rodent-borne viruses that cause 2 severe diseases in humans: hantavirus cardiopulmonary syndrome in the Americas and hemorrhagic fever with renal syndrome (HFRS) in Eurasia. HFRS causes ≈150,000–200,000 hospitalizations each year throughout the world (*1*). Puumala virus (PUUV), which is spread in large areas of Europe, causes a milder form of HFRS called nephropathia epidemica (*2*). Since 1989, when the disease became notifiable in Sweden, the largest number of cases was reported during 2007 (2,195) compared with a median 207.5 cases during 1990–2007 (M. Hjertqvist, pers. comm.). The mean incidence of nephropathia epidemica in the 4 northernmost county councils in Sweden was as high as 225.5/100,000 in 2007 (*3*).

PUUV is carried and maintained by infected bank voles (*Myodes glareolus*); transmission is believed to occur by inhalation of virus-containing, aerosolized, rodent excreta (*4*). Infectious PUUV has been detected in saliva, urine, and feces from experimentally infected colonized bank voles (*5*), and excreted PUUV is infectious for up to 12–15 days outside the host (*6*). However, the relative importance of saliva, urine, and feces in transmission of PUUV between bank voles or from bank voles to humans and how levels of virus change over time in different excretions are not known.

In this study, we used real-time reverse transcription–PCR (RT-PCR) to measure levels of shed viral RNA in saliva, urine, and feces of subcutaneously inoculated bank voles until they were killed at day 133 postinfection (PI). To evaluate possible transmission routes for PUUV, we investigated infectivity of different excretions and used a subset of viral RNA–positive saliva, urine, and feces samples to intranasally inoculate virus-negative bank voles.

## Materials and Methods

### Animals and Virus

Colonized bank voles were maintained in separate cages in biologic safety isolators with food and water provided ad libitum. All handling of animals was in compliance with guidelines of the Swedish Institute for Infectious Disease Control, Stockholm, Sweden. The PUUV strain Kazan wild type (PUUV Kazan-wt) (*7*,*8*) was used for subcutaneous inoculation of bank voles, and Vero E6 cell line–adapted PUUV strain Kazan (PUUV Kazan-E6) (*7*) was used in inhibition experiments and as a positive control in the real-time RT-PCR.

### Subcutaneous Inoculation and Sample Handling

Bank voles were subcutaneously inoculated with ≈200 bank vole 50% infectious doses of PUUV Kazan-wt diluted in Hanks balanced salt solution medium (Invitrogen, Paisley, Scotland). Animals were sampled for saliva, urine, and feces on days 0, 1, 2, 3, 4, 8, 9, 11, 14, 16, 21, 28, 35, 42, 49, 56, 63, 70, 77, 84, 91, and 133 PI. Serum samples were obtained on day 21 and at the termination of the experiment (day 133 PI). Some animals did not survive until day 133, but they were sampled by using the same procedures until time of death.

Saliva was collected by gently rotating a moistened cotton swab in the mouth of the bank vole. The cotton swab was subsequently placed in a cryotube containing 500 μL dilution medium (Hanks balanced salt solution medium containing 2% HEPES [Invitrogen], 2% fetal calf serum [Sigma-Aldrich, St. Louis, MO, USA], and 1% penicillin-streptomycin [Sigma]). Urine was collected by grasping the scruff of the neck of the animal and holding it over a petri dish to cause urination, as described by Botten et al. (*9*). Urine samples were stored in cryotubes. For feces sampling, bank voles were placed in separate containers until feces could be collected and transferred into cryotubes. All saliva, urine, and feces samples were stored at –70°C until analyzed. Serum samples were stored at –20°C until analyzed for antibodies to PUUV by ELISA.

### Intranasal Inoculation

Intranasal inoculation was performed by using subsets of the PUUV RNA–positive excretion samples from subcutaneously inoculated bank voles (pooled saliva samples from bank voles no. 6 [day 21] and no. 7 [day 21]; pooled urine samples from bank voles no. 10 [day 16], no. 1 [day 21], no. 8 [day 28], and no. 7 [day 14]; and feces suspension from bank vole no. 3 [day 21]). A total of 5 μL of saliva, urine, or feces suspension was delivered to each nostril of 14 anesthetized bank voles. Saliva, urine, and feces were administered to groups of 4, 5, and 5 bank voles, respectively. These intranasally inoculated bank voles were sampled for saliva, urine, and feces on days 0, 6, 14, 21, 26, 35, and 42 PI, and a subset of these samples was tested for PUUV RNA by real-time RT-PCR. Animals were bled at days 21 and 42 PI and then humanely killed. All handling of samples was performed as described for the subcutaneous inoculation experiment.

### ELISA

To confirm PUUV infection of the animals, a PUUV-nucleocapsid immunoglobulin (Ig) G ELISA (*10*) was performed by using serum from day 21 PI for the subcutaneous inoculation experiment, and from days 21 and 42 PI for the intranasal inoculation experiment. Briefly, 1 μg/mL of rKAZ (*Escherichia coli*–expressed recombinant PUUV Kazan) was coated on a 96-well plate. After washing and blocking, samples to be tested were added to the plate in duplicate at dilution of 1:200. After washing, alkaline phosphatase–conjugated goat anti-mouse IgG (Jackson Immuno Research, West Grove, PA, USA) was added to the plate. The plate was washed again and *p*-nitrophenyl phosphate (Sigma-Aldrich) substrate was added; the optical density was determined at 405–620 nm.

### Extraction of RNA

Viral RNA was extracted by using the Ex-tract DNA/RNA Extraction Kit (Severn Biotech Ltd., Kidderminster, UK) with procedures described by Boom et al. (*11*) with minor modifications. For saliva samples, 100 μL of sample was transferred into a new tube containing 100 μL of dilution medium, 20 μL of silica particles, and 1 mL of L6 buffer. Urine samples were centrifuged at 1,800 × *g* for 5 min, and 20 μL of supernatants was transferred to a new tube containing 180 μL of dilution medium, 20 μL of silica particles, and 1 mL of L6 buffer. Approximately 50 mg of fecal sample was homogenized in 600 μL of phosphate-buffered saline (PBS) and centrifuged at 1,800 × *g* for 5 min. A total of 200 μL of supernatant was transferred to a tube containing 20 μL of silica particles and 1 mL of L6 buffer.

Tubes were vortexed for 10 s and incubated for 15 min at room temperature on a shaker. After centrifugation at 15,700 × *g* for 45 s, pellets were washed twice with 1 mL of L2 buffer, twice with 1 mL of 70% ethanol, and once with 1 mL of acetone. After acetone removal, the pellet was dried at 56°C for 5–10 min, dissolved in 49 μL of RNase-free water (Invitrogen) and 1 μL of RNaseOUT (Invitrogen), and incubated at 56°C for 15 min. After centrifugation at 15,700 × *g* for 4 min, the supernatant was transferred into a new tube and immediately analyzed for viral RNA by using real-time RT-PCR. PUUV Kazan-E6 (30,000 focus-forming units [FFU]/mL) was used as a positive control.

### Real-Time RT-PCR

A real-time RT-PCR targeting the small segment of the PUUV genome was performed by using the QuantiTect Probe RT-PCR Kit (QIAGEN, Hilden Germany). The reaction consisted of 1× QuantiTect Probe RT-PCR master mixture, 300 nmol/L forward primer 983F (5′-GTGCACCAGATCGGTGTCC-3′) (Invitrogen) (*12*), 900 nmol/L reverse primer 1038R (5′-CAATTCAGCCATCCCAGCA-3′) (Invitrogen) (*12*), 150 nmol/L TaqMan MGB probe 1003T (5′-CCTACATGCATTTATG-3′) (Applied Biosystems, Warrington, UK) (*12*), 0.25 μL of QuantiTect RT mixture, 5 μL of sample RNA (corresponding to RNA from 2 μL urine, ≈5 mg feces, or 10 μL oral swab suspension), and RNase-free water (Invitrogen) to give a final volume of 25 μL. A 96-well plate (Bio-Rad Laboratories, Hercules, CA, USA) was used, and PCR thermal cycling was performed by using an iCycler (Bio-Rad Laboratories) with the following cycling conditions: 50°C for 30 min and 95°C for 15 min, followed by 45 cycles at 94°C for 15 s and 60°C for 1 min. All samples were tested in duplicate.

### Evaluation of Real-Time RT-PCR Inhibition by Bank Vole Excretions

Samples of saliva, urine, and feces from uninfected bank voles were prepared similarly to samples from infected bank voles. Saliva was diluted twice in dilution medium, urine was diluted 10 times in dilution medium, and feces was prepared as an 8% suspension in PBS. Uninfected samples were spiked with PUUV Kazan-E6 at 10-fold serial dilutions ranging from 30 to 30,000 FFU/mL. Dilution medium and PBS were used as controls. All samples were extracted and analyzed in duplicate by using real-time RT-PCR.

## Results

### Inhibition of PUUV Real-Time RT-PCR by Feces

**Figure 1:**
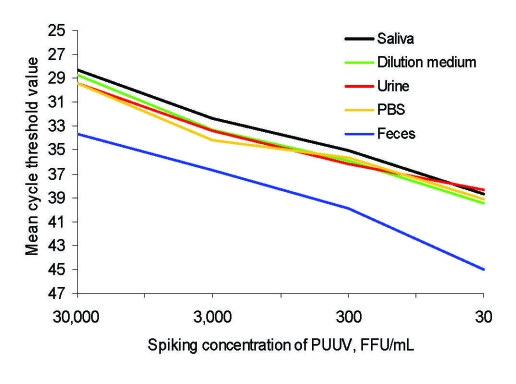
Inhibition of Puumala virus (PUUV) real-time reverse transcription–PCR by feces, but not saliva or urine, of bank voles. Mean cycle threshold values are shown for different solutions spiked with a cell line–adapted PUUV. Cycle threshold values of negative samples were set at 45. PBS, phosphate-buffered saline; FFU, focus-forming units.

Urine and saliva samples showed cycle threshold values similar to dilution medium and the PBS control for all viral dilutions. However, feces samples spiked with PUUV Kazan-E6 showed cycle threshold values ≈3–6 cycles above control values (Figure 1), which has been shown to correspond to 10–100× lower detection of RNA for all viral dilutions (*13*).

### Kinetics of Excreted PUUV RNA from Subcutaneously Inoculated Bank Voles

Ten colonized male bank voles were subcutaneously inoculated with PUUV Kazan-wt. All bank voles seroconverted, as shown by an IgG ELISA that used serum samples obtained from animals at day 21 PI. Four bank voles did not survive until day 133; bank voles no. 4 and 9 died after being anesthetized on day 21 PI, and bank voles no. 5 and 10 died of unknown reasons on 112 and 35 days PI, respectively.

**Figure 2:**
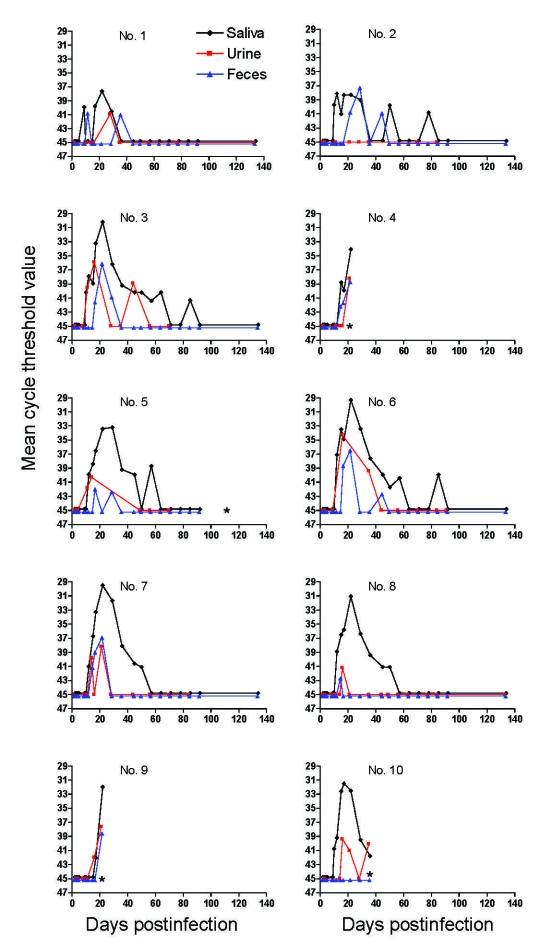
Detection of Puumala virus (PUUV) RNA by real-time reverse transcription–PCR in saliva, urine, and feces of bank voles subcutaneously inoculated with PUUV strain Kazan wild type. Cycle threshold values of negative samples were set at 45. *, bank voles 4 and 9 died on day 21 postinfection and bank voles 5 and 10 died on days 112 and 35 postinfection, respectively.

Viral RNA was detected in subsets of saliva, urine, and feces samples (Figure 2, Table 1). Cycle threshold values of negative samples were set at 45. Levels of excreted PUUV RNA peaked on days 11–28 PI for saliva, 14–28 PI for urine, and 11–28 PI for feces. The earliest and latest detection of PUUV RNA was found for saliva on days 8 and 84 PI, compared with 11 and 44 days PI for urine and feces. One animal (no. 2) was PUUV RNA negative in all urine samples, and 1 animal (no. 10, which died on day 35 PI) was negative in all feces samples (Table 1). Viral RNA was detected in serum from 5 of the 6 surviving animals at day 133.

**Table 1:** Detection of PUUV RNA over time in excreta from bank voles subcutaneously inoculated with PUUV strain Kazan wild type*

Bank vole no.	Excretion type	Detection of PUUV RNA in excreta by time, d																				
		1	2	3	4	8	9	11	14	16	21	28	35	44	49	56	63	70	77	84	91	133
1	Saliva	–	–	–	–	+	–	–	–	–	+	+	–	–	–	–	–	–	–	–	–	–
	Urine	NT	–	NT	NT	NT	NT	–	–	–	NT	+	–	NT	NT	–	NT	NT	–	NT	NT	–
	Feces	–	–	–	–	–	–	+	–	–	–	–	+	–	–	–	–	–	–	–	–	–
2	Saliva	–	–	–	–	–	+	+	+	+	+	+	–	–	+	–	–	–	+	–	–	–
	Urine	–	NT	–	NT	NT	NT	–	NT	NT	–	–	–	NT	NT	NT	NT	–	NT	–	NT	NT
	Feces	–	–	–	–	–	–	–	–	–	+	+	–	+	–	–	–	–	–	–	–	–
3	Saliva	–	–	–	–	–	+	+	+	+	+	+	+	+	+	+	+	–	–	+	–	–
	Urine	–	–	NT	NT	–	NT	+	NT	+	NT	–	–	+	NT	NT	NT	–	NT	NT	NT	NT
	Feces	–	–	–	–	–	–	–	–	+	+	+	–	–	–	–	–	–	–	–	–	–
4†	Saliva	–	–	–	–	–	–	–	+	+	+											
	Urine	–	–	NT	NT	NT	NT	–	–	–	+											
	Feces	–	–	–	–	–	–	–	+	+	+											
5‡	Saliva	–	–	–	–	–	–	+	+	+	+	+	+	+	–	+	–	–	–	–	–	
	Urine	NT	–	NT	–	NT	NT	+	+	NT	NT	NT	NT	NT	–	–	NT	–	NT	NT	NT	
	Feces	–	–	–	–	–	–	–	–	+	–	+	–	–	–	–	–	–	–	–	–	
6	Saliva	–	–	–	–	–	–	+	+	+	+	+	+	+	+	+	–	–	-	+	–	–
	Urine	–	NT	–	NT	NT	–	NT	NT	+	NT	NT	+	–	NT	–	NT	–	NT	-	–	NT
	Feces	–	–	–	–	–	–	–	–	+	+	–	–	+	–	–	–	–	–	–	–	–
7	Saliva	–	–	–	–	–	–	+	+	+	+	+	+	+	+	–	–	–	–	–	–	–
	Urine	–	NT	NT	NT	NT	–	–	+	+	+	–	NT	–	NT	–	NT	–	NT	–	NT	NT
	Feces	–	–	–	–	–	–	–	+	+	+	–	-	–	–	–	–	–	–	–	–	–
8	Saliva	–	–	–	–	–	–	+	+	+	+	+	+	+	+	–	–	–	–	–	–	–
	Urine	–	–	–	NT	NT	–	–	–	+	–	NT	–	–	–	–	–	NT	–	–	NT	–
	Feces	–	–	–	–	–	–	–	+	–	–	–	–	–	–	–	–	–	–	–	–-	–
9†	Saliva	–	–	–	–	–	–	–	–	+	+											
	Urine	NT	NT	–	–	–	–	–	NT	+	+											
	Feces	–	–	–	–	–	–	–	–	–-	+											
10§	Saliva	–	–	–	–	–	+	+	+	+	+	+	+									
	Urine	NT	NT	–	NT	NT	NT	NT	–	+	+	–	+									
	Feces	–	–	–	–	–	–	–	–	–	–	–	–									

*PUUV, Puumala virus; –, negative; +, positive, NT, not tested (no or insufficient urine volume). †Died on day 21 postinfection. ‡Died on day 112 postinfection. §Died on day 35 postinfection.

### Intranasal Transmission of PUUV by Bank Vole Saliva, Urine, and Feces

We tested whether RNA-positive excretion samples also contained infectious virus and whether intranasal inoculation was a possible route of infection for all types of excretions. A subset of the PUUV RNA–positive urine, feces, and saliva samples collected from subcutaneously inoculated bank voles was administered intranasally to 14 virus-negative female bank voles. Seven (2/4 given saliva, 2/5 given urine, and 3/5 given feces) of 14 intranasally inoculated bank voles seroconverted (Table 2).

**Table 2:** Infection of bank voles with PUUV after intranasal inoculation with saliva, urine, and feces samples*

Inoculum	Bank vole no.	Seropositivity			PUUV RNA in excreta by time, d†		
		Day 21 PI	Day 42 PI		Saliva	Urine	Feces
Saliva	1	–	+		NT	NT	NT
	2	–	–		NT	NT	NT
	3	–	–		NT	NT	NT
	4	+	+		0, 6, 14, **19, 26, 35, 42**	0, **19, 26, 42**	0, 6, 14, **19, 26, 35, 42**
Urine	5	–	+		NT	0, 24, **26, 28**	NT
	6	–	–		NT	NT	NT
	7	–	+		0, 6, 14, 19, 26, **35, 42**	0, 14, 19, 35	0, 6, 14, 19, 26, 35, **42**
	8	–	–		NT	NT	NT
	9	–	–		NT	NT	NT
Feces	10	–	+		NT	6, 14, 24, **26, 35, 42**	NT
	11	–	–		NT	NT	NT
	12	–	+		NT	0, 24	NT
	13	–	+		0, 14, 19, 26, **35, 42**	6, 19, 24	0, 6, 19, 26, **35, 42**
	14	–	–		NT	NT	NT

*PUUV, Puumala virus; PI, postinfection; –, negative; +, positive; NT, not tested. †Numbers indicate days PI for tested excretions. Days with positive excretions are shown in **boldface**.

Saliva, urine, and feces samples were obtained from the 14 intranasally inoculated animals and tested by real-time RT-PCR. PUUV RNA was detected in subsets of saliva, urine, and feces samples from all 3 groups (Table 2).

## Discussion

We have shown in controlled experimental conditions how levels of shed PUUV RNA change over time in saliva, urine, and feces from PUUV-infected bank voles. All 3 excretions can transmit virus to other bank voles when administered intranasally, which suggests that all 3 excretion pathways can function as natural transmission routes between bank voles and from bank voles to humans.

In previous studies on PUUV, experimentally infected bank voles seem to excrete infectious virus for a limited time after infection (*5*,*14*). This finding is consistent with our real-time RT-PCR data. We observed clear peaks of shed viral RNA in saliva, urine, and feces preceded and followed by levels below detection limits. Viral RNA was detected in blood of 5 of 6 surviving animals on day 133, which suggested that persistently infected bank voles do not normally shed virus during the entire course of infection. Levels of excreted viral RNA decreased below the detection level in some animals, but RNA was detected in subsequent samples (Figure 2).

Similar patterns have been observed for Sin Nombre virus (SNV)–infected deer mice (*Peromyscus maniculatus*). Botten et al. reported an initial peak in SNV RNA levels in lung samples at 21 days PI, followed by a second peak at 60 days PI (*15*). In another report on SNV, Kuenzi et al. found a variation in PCR positivity of blood samples from wild-caught deer mice (*16*). These authors suggested 2 interpretations of the results: either that viral RNA is consistently present in the blood but is near the limits of PCR detectability or viral RNA reappears in blood as a consequence of unknown physiologic events. We believe that similar interpretations can be made concerning levels of PUUV RNA in bank vole excretions. Whether levels of excreted PUUV change as a consequence of external factors, e.g., cold temperatures or social stress, remain to be shown.

A problem when working with biologic material combined with PCR techniques is the effect of inhibitory substances; several inhibitory components in feces have been identified, such as bile salts and polysaccharides (*17*). In the spiking experiments, saliva and urine showed no PCR inhibition because results for excretions were comparable to those of dilution medium and PBS (similar cycle threshold values). In contrast, 10–100× less viral RNA was recovered from spiked feces samples (Figure 1), which indicated that more virus was shed in bank vole feces than we were able to detect. We conclude that saliva contained higher levels of viral RNA than urine did because saliva samples were ≈10–20× more diluted than the urine samples but still showed lower cycle threshold values.

Although real-time RT-PCR is an effective method for measuring levels of RNA, it does not necessarily measure the presence of infectious virions. We therefore tested a subset of real-time RT-PCR–positive excretion samples for infectious virus. Different methods can be used to detect infectious hantavirus and potential transmission routes. Bernshtein et al showed that more bank voles were infected when injected with lung suspension from PUUV-positive bank voles than after intercage transmission (*14*). Injection shows if an excretion contains infectious virus, but in nature a similar event will occur only when saliva is transferred by biting. Especially for urine and feces, intranasal inoculation probably resembles natural transmission. Bank vole saliva, urine, and feces are infectious when injected intramuscularly into virus-negative bank voles (*5*). We show that saliva, urine, and feces are also infectious when given intranasally, which indicates that PUUV in bank vole saliva can be transferred not only by biting. Intranasal inhalation of saliva may also involve ingestion, which may also be a viable route of infection. Ingestion could occur when several bank voles share a common food source. Hooper et al. have recently shown that Andes hantavirus is infectious to hamsters when administered by intragastric injection and speculate that ingestion of contaminated material might be a mode of transmission to humans (*18*).

We have shown that intranasal inoculation of saliva, urine, or feces enables subsequent detection of viral RNA in all types of excretions, which indicates that virions excreted by different routes do not show restricted tropism for particular tissues. When we analyzed serum obtained on day 42 PI from intranasally inoculated bank voles by ELISA, 7 of 14 had seroconverted (Table 2). Only 1 of the animals was positive at day 21 PI (Table 2). This late seroconversion in bank voles may have been caused by relatively low doses of virus in bank vole saliva, urine, and feces samples used for intranasal inoculation. We believe that this information will be useful in future vaccine and infection studies because it indicates that a low level of hantavirus might not induce seroconversion until after 21 day PI. It would be useful to investigate whether a low dose of hantavirus inoculum can induce seroconversion after 42 day PI.

To better evaluate and predict risk for human hantavirus infections, information on factors associated with occurrence and transmission of hantavirus in natural rodent populations is needed. It has been assumed that rodent behavior is required for maintenance of PUUV in the natural reservoir because PUUV infection in relation to bank vole demography shows nonrandom transmission patterns (*19*). PUUV stability outside the host likely plays a role in transmission to other rodents and in the number of human cases (*20*). Hantaviruses have been shown to be stable ex vivo, and Hantaan hantavirus can infect cell culture after being stored for as long as 96 days in medium at 4°C (*21*). Furthermore, PUUV is infectious for bank voles for up to 12–15 days in contaminated cage bedding (*6*). How different excretions contribute to virus stability in the environment and what implications this might have on direct versus indirect transmission among rodent reservoirs remain to be shown. The role of different excretions in transmission of PUUV may vary with the age and density of bank voles and the season. Hypothetically, shedding in saliva might be more efficient for virus transmission in male bank voles living in a high-density area during mating season, when many fights occur. In contrast, shedding in feces, which may provide the virus with a more stable environment, may play a more dominant role in transmission in a low-density area during fall or winter.

In conclusion, we studied levels of PUUV RNA in excretions of infected bank voles over a period of 4.5 months. We have shown that bank vole saliva, urine, and feces can cause infection when inhaled by other bank voles, which indicates that all 3 excretions can transfer virus to humans.
